# Microbiota–Mediator–Host Signaling Networks in Metabolic Syndrome: From Mechanistic Insights to Therapeutic Targeting

**DOI:** 10.3390/microorganisms14091865

**Published:** 2026-08-22

**Authors:** Xinyi Zhuang, Xiang Li, Zhengle Yang, Xiahong Dai

**Affiliations:** 1 School of Medicine, Zhejiang Chinese Medical University, Hangzhou 310000, China; xinyizhuang@zcmu.edu.cn (X.Z.); 18257271537@163.com (X.L.); yangzhengle2025@163.com (Z.Y.); 2 Department of Infectious Diseases, Shulan (Hangzhou) Hospital, Shulan International Medical College, Zhejiang Shuren University, Hangzhou 310000, China

**Keywords:** metabolic syndrome, gut microbiota, gut microbiota-associated mediators, microbiota–mediator–host signaling networks, gut microbiota-targeted therapeutic

## Abstract

Metabolic syndrome (MetS) represents a growing global health burden characterized by obesity, insulin resistance, dyslipidemia, and hypertension. Increasing evidence suggests that gut microbiota-associated mediators may serve as signaling intermediates involved in host metabolic regulation. However, the mechanisms by which these mediators interact with host signaling pathways and influence metabolic responses remain incompletely understood. This review summarizes current advances in gut microbiota-associated mediators, focusing on short-chain fatty acids, bile acids, lipopolysaccharide, trimethylamine N-oxide, and branched-chain amino acids. We discuss their interactions with host metabolic and inflammatory pathways, including pathways implicated in FFAR2/3-mediated signaling, FXR/TGR5 signaling, TLR4/NF-κB-mediated inflammatory signaling, and mTORC1-associated nutrient-sensing. Furthermore, we propose a microbiota–mediator–host signaling network framework as an emerging conceptual model to integrate these molecular interactions and highlight the utility of multi-omics approaches in characterizing complex microbiota–host communication. Despite mechanistic advances, substantial challenges remain, including heterogeneous microbial signatures across populations, limited causal evidence, inter-individual variability in therapeutic responses, and barriers to clinical translation of microbiota-targeted interventions. A better understanding of microbiota-associated signaling networks may provide new insights into metabolic regulation and contribute to the rational development of microbiota-targeted strategies that complement established lifestyle interventions for MetS management.

## 1. Introduction

Metabolic syndrome (MetS) has emerged as a major global health challenge, primarily driven by chronic positive energy balance, excessive caloric intake, and physical inactivity [1,2]. The prevalence of MetS is increasing due to continued socioeconomic development, westernized lifestyles, and an aging global population [3]. This trend imposes a substantial burden on healthcare systems and global chronic disease management [1,3]. Currently, treatment strategies for MetS remain largely centered on lifestyle interventions, including dietary modification, increased physical activity, and pharmacological combinations targeting hyperglycemia, hypertension, and dyslipidemia [4]. Although these approaches remain the cornerstone of MetS management and have been shown to improve metabolic health and modulate gut microbial profiles [5], their long-term effectiveness may be influenced by heterogeneous individual responses, challenges in sustained adherence, and the multifactorial nature of metabolic dysfunction [6,7]. Therefore, there is a need to develop adjunctive therapeutic strategies to improve MetS management.

Beyond traditional metabolic risk factors, increasing attention has been directed toward biological regulatory processes that connect environmental exposures with host metabolic dysfunction. Among these processes, the gut microbiome represents an important regulatory intermediate linking environmental factors, microbial functions, and host metabolic physiology [8,9]. Through its diverse metabolic capacities and interactions with the host, the gut microbiome may contribute to metabolic homeostasis. Growing evidence suggests that gut dysbiosis is frequently observed in individuals with MetS, involving alterations in microbial diversity, community composition, and metabolic functionality [9]. Thus, gut dysbiosis and microbiota-associated mediators are increasingly recognized as potential regulatory intermediaries within the multifactorial landscape of MetS, linking lifestyle-derived metabolic stressors with alterations in microbial metabolite functions and host metabolic and inflammatory signaling responses.

Importantly, microbial functional alterations are not merely reflected by changes in community composition, but also by the production and transformation of bioactive molecules that influence host physiology. These microbiota-associated mediators provide a functional link between microbial activities and host metabolic regulation, serving as potential intermediates in host–microbe communication [9,10]. These mediators include short-chain fatty acids (SCFAs), bile acids (BAs), lipopolysaccharide (LPS), trimethylamine N-oxide (TMAO), and branched-chain amino acids (BCAAs) [9,10]. However, the biological effects of these mediators are context-dependent and shaped by interactions among microbial activity, dietary factors, and host metabolic status [9,10]. Through these complex interactions, microbiota-associated mediators may modulate host energy metabolism, immunological responses, and inflammatory signaling pathways [11,12,13]. As nutrient-sensing signals, metabolic regulators, and inflammatory mediators, these mediators have been implicated in both metabolic homeostasis and metabolic dysfunction [9,10]. For example, SCFAs and BAs have been reported to contribute to metabolic homeostasis by regulating host metabolic signaling pathways and maintaining intestinal barrier integrity [14,15]. In contrast, LPS and TMAO tend to be related to inflammation, cardiometabolic dysfunction, and metabolic endotoxemia [16,17]. BCAAs have been implicated in insulin resistance through metabolic signaling pathways and nutrient sensing [18].

These effects involve interactions with host signaling pathways and receptors. Therefore, increasing attention has been directed toward elucidating how microbiota-associated mediators integrate microbial functions with host metabolic signaling networks in metabolic diseases [9,10]. Although substantial progress has been made in characterizing gut microbiota alterations and microbiota-associated mediators in MetS, a system-level framework describing how these mediators collectively interact with host signaling pathways remains lacking. Recent advances in multi-omics approaches have provided new opportunities for integrating microbial functions, metabolite profiles, and host responses [19,20]. Improved understanding of microbiota–host interactions has provided a rationale for exploring microbiota-targeted interventions, including probiotics, prebiotics, fecal microbiota transplantation (FMT), and live biotherapeutic products (LBPs) [9,10]. However, their clinical translation remains challenging due to heterogeneous efficacy, inter-individual variability, and limited understanding of the underlying mechanisms [21,22,23]. Therefore, a comprehensive understanding of how gut microbiota-associated mediators interact with host signaling pathways is important for clarifying their roles in microbiota–host metabolic communication. Such integrated insights may provide a basis for developing targeted microbiota-based adjunctive strategies for MetS management.

This review systematically summarizes current knowledge on gut microbiota and their associated mediators in MetS and discusses emerging microbiota-targeted therapeutic strategies within the context of a microbiota–mediator–host signaling network.

## 2. Gut Microbiota Dysbiosis and Metabolic Alterations in MetS

**Figure 1:**
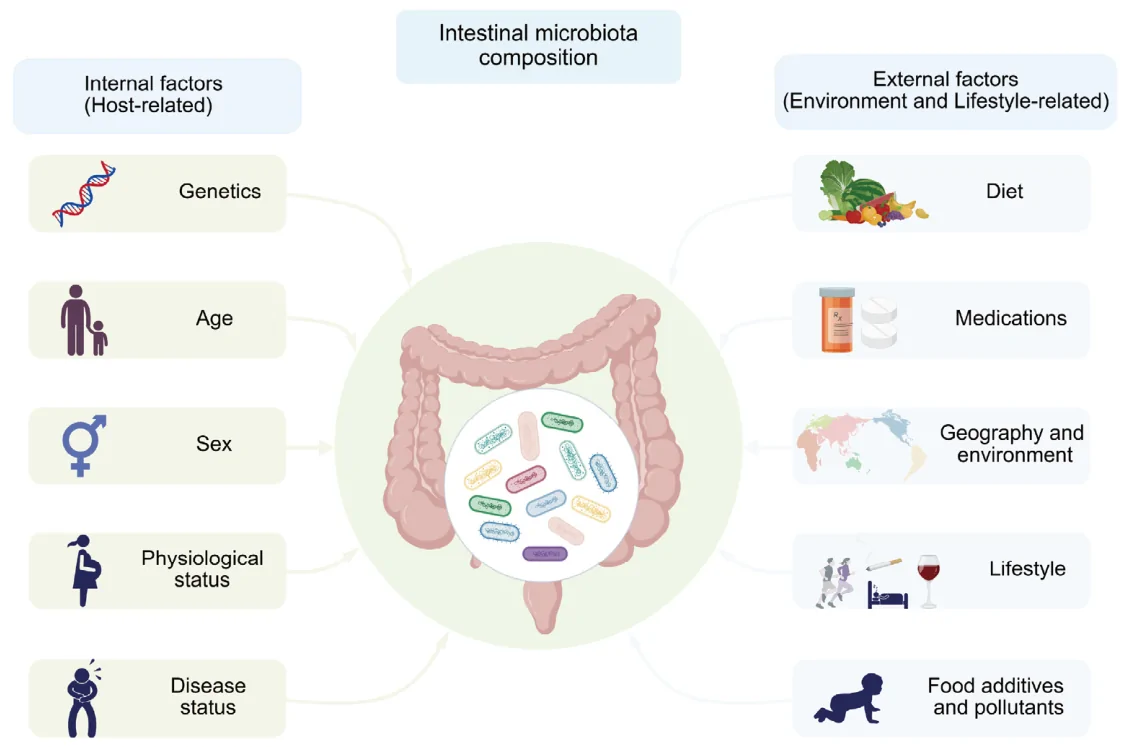
Endogenous and exogenous factors affecting gut microbiota composition. Created with BioGDP.com [27].

MetS is a multifactorial metabolic disorder shaped by genetic susceptibility, lifestyle factors, environmental exposures, and host metabolic status [1,3]. Increasing evidence suggests a complex association between gut microbiota alterations and MetS-related metabolic abnormalities [9]. Gut microbiota composition is shaped by complex interactions among host factors, dietary exposures, lifestyle behaviors, and environmental conditions, including age, sex, genetic background, disease status, and geographical location [24,25,26] (Figure 1).

Altered microbial diversity is one of the commonly reported features of gut microbiota dysbiosis in individuals with MetS [9]. α-diversity reflects within-sample microbial diversity, including species richness and evenness, whereas β-diversity describes differences in microbial community composition among samples [24]. Reduced α-diversity has been frequently reported in individuals with MetS, although findings vary across different cohorts [9]. Beyond changes in microbial diversity, alterations in microbial community composition have also been observed in individuals with MetS. Reported microbial alterations have included changes in the Firmicutes/Bacteroidetes (F/B) ratio [9,28], altered abundance of taxa associated with metabolic health, and enrichment of potential pathobionts [29]. However, taxonomic signatures reported across different cohorts remain inconsistent, and their biological relevance to MetS remains incompletely understood [9,30]. Given the substantial inter-individual and cohort-level variability, these findings should be interpreted as context-dependent associations rather than universal microbial signatures [9]. Table 1 summarizes frequently reported microbial alterations and representative taxa across MetS-related metabolic phenotypes.

Despite considerable heterogeneity in microbial profiles associated with metabolic disorders, several recurring patterns have been observed across MetS-related phenotypes [9,45]. Reduced microbial diversity and gene richness have been frequently reported in obesity, type 2 diabetes, and other metabolic disorders compared with metabolically healthy individuals [46]. Beyond diversity alterations, MetS-related disorders have also been associated with microbial community changes linked to low-grade inflammation and metabolic endotoxemia, although the specific taxa involved vary considerably among populations and disease contexts [9,16].

Opportunistic pathogens and pro-inflammatory microbial communities are frequently observed in individuals with metabolic disorders, despite the specific taxa involved varying considerably among populations and disease patterns [9,16,45]. These microbial alterations have been associated with impaired intestinal barrier integrity and facilitate systemic inflammatory responses, potentially involving microbiota-associated mediators [16].

However, taxonomic associations alone provide limited insight into the functional consequences of gut microbiota alterations. Significant variability exists among cohorts, particularly for taxa involved in SCFA metabolism [9,47]. For example, some studies have reported an enrichment of specific Firmicutes taxa that have been associated with carbohydrate fermentation and energy-harvesting capacity in obese individuals [28], whereas other cohorts have reported depletion of butyrate-producing taxa across metabolic diseases [48]. These apparently conflicting findings highlight the limitations of interpreting microbial alterations solely based on taxonomic composition. They may result from functional heterogeneity within bacterial taxa, dietary variation, host metabolic state, and methodological variation across studies [9,34].

Importantly, distinct microbial community structures may converge on similar metabolic characteristics through functional redundancy [49]. Different microbial taxa can contribute to overlapping metabolic pathways, potentially resulting in similar functional outputs or microbiota-associated mediator profiles despite differences in taxonomic composition [24,50]. Therefore, taxonomic signatures identified in one population may not necessarily represent universal features across other populations [9,51].

Despite considerable heterogeneity in microbial composition among individuals, recurring patterns in gut microbiota-associated mediator profiles have been reported across metabolic phenotypes, including obesity, IR, dyslipidemia, and hypertension. These include changes in SCFA metabolism, dysregulated BA profiles, increased LPS exposure, elevated TMAO levels, and altered BCAA metabolism [45]. However, most current evidence remains associative, and the causal contributions of gut microbial activity to host metabolic regulation remain incompletely defined [9,45]. Given that microbiota-associated functions and mediator production are shaped by dietary, host, and environmental factors [52], integrating microbial taxonomic profiles with microbiota-associated mediators may provide a more comprehensive framework for understanding microbiota–host metabolic interactions in MetS [10].

## 3. Gut Microbiota-Associated Mediators

Chronic positive energy balance, excessive caloric intake, and reduced physical activity represent major drivers of MetS [3]. These factors can also shape gut microbial ecosystems and influence their functional activity [8]. Microbiota-associated mediators influenced by these factors may act as regulatory intermediates between lifestyle-related metabolic stressors and host metabolic pathways and are involved in the regulation of inflammatory responses, glucose homeostasis, lipid metabolism, and energy balance [9,10].

### 3.1. Short-Chain Fatty Acids

SCFAs, including acetate, propionate, and butyrate, are major microbial fermentation products of dietary fibers and key mediators of host–microbiota metabolic communication [47,53]. Several bacterial taxa, including *Faecalibacterium*, *Roseburia*, *Eubacterium*, and *Anaerostipes*, have been associated with intestinal butyrate production. *Bifidobacterium* mainly generates acetate, while *Bacteroides* have been associated with acetate and propionate [54]. However, metabolite production is not solely determined by individual taxa, as functional redundancy among microbial communities and substrate availability may influence SCFA profiles. Despite this taxonomic variability, SCFAs represent an important functional output of gut microbial metabolism and serve as signaling molecules linking microbial activity with host physiological responses. SCFAs influence intestinal barrier integrity, energy metabolism, and immune responses through multiple mechanisms [47].

Functionally, butyrate serves as the main energy source for colonocytes [55], stabilizes HIF-1α protein, upregulates ZO-1, Occludin, and Claudin-1 levels, while reducing Claudin-2 expression, thereby reducing intestinal epithelial permeability and limiting the translocation of LPS and TMAO into the bloodstream [55,56]. In contrast, acetate and propionate enter the bloodstream and participate in the overall metabolic regulation involving the liver, skeletal muscles, and adipose tissues [57,58].

At physiological concentrations, propionate and butyrate can function as histone deacetylase (HDAC) inhibitors, thereby modulating inflammatory responses and contributing to metabolic homeostasis [57,58]. Additionally, SCFAs can activate free fatty acid receptor 2/3 (FFAR2/3) [59], decrease intracellular cAMP, and reduce PKA-mediated AMPK inhibition [60]. Once activated, AMPK suppresses excessive inflammatory responses by preventing NOD-like receptor family pyrin domain-containing protein 3 (NLRP3) inflammasome activation, inhibiting nuclear factor-κB (NF-κB) signaling, and regulating macrophage immunometabolism [61,62]. Moreover, SCFAs can stimulate FFAR2/3 on enteroendocrine cells, promoting the secretion of peptide YY (PYY) and glucagon-like peptide-1 (GLP-1) [63]. GLP-1 impacts glucose homeostasis [64], whereas PYY contributes to satiety and energy balance [65].

Animal studies provide significant evidence for the role of gut microbiota-associated SCFAs in metabolic control. In animal models, FFAR2 signaling has been implicated in body-weight regulation, with activation conferring protection against diet-induced obesity, whereas loss of function increases the risk of obesity [60]. Compared with conventionally raised mice, germ-free mice exhibit lower intestinal SCFA levels and altered susceptibility to diet-induced obesity [66]. Additionally, transplanting gut microbiota into germ-free recipients can alter SCFA production and influence adiposity [67], suggesting that gut microbiota-mediated changes in SCFA availability may influence host energy metabolism under specific experimental conditions.

Interestingly, the relationship between SCFAs and metabolic health appears to be context-dependent [53,68]. Although SCFAs have been associated with improved insulin sensitivity, reduced inflammation, and enhanced intestinal barrier function, some studies have found that individuals with obesity have higher fecal SCFA concentrations [47]. These findings indicate that, under certain metabolic circumstances, increased microbial fermentation may reflect altered energy harvest efficiency and metabolic adaptation associated with obesity [28]. Consequently, SCFA concentrations, tissue distribution, and host metabolic state likely influence their metabolic effects. Further research is needed to better understand how host metabolic status, microbial composition, and environmental factors shape the context-dependent effects of SCFAs on host metabolic regulation.

### 3.2. Bile Acids

BAs are important gut microbiota-associated mediators involved in the regulation of lipid, glucose, and energy metabolism [69]. Primary BAs synthesized in the liver are further modified by gut microbial communities through bile acid-transforming processes, particularly involving microbial communities with bile salt hydrolase (BSH) activity and 7α-dehydroxylation capacity [70]. Members of the genera such as *Bacteroides*, *Bifidobacterium*, and *Lactobacillus* have been reported to contribute to bile acid deconjugation through BSH activity, whereas Clostridia-related taxa, particularly *Clostridium scindens*, mediate 7α-dehydroxylation reactions that convert primary BAs into secondary BAs, including deoxycholic acid (DCA) and lithocholic acid (LCA) [70]. These microbiota-associated bile acid metabolites, including DCA and LCA, can act as endogenous ligands for Takeda G protein-coupled receptor 5 (TGR5), whereas specific BAs such as chenodeoxycholic acid (CDCA) are potent farnesoid X receptor (FXR) ligands. Through FXR and TGR5 signaling, bile acid profiles influence host metabolic regulation [71].

FXR and TGR5 mediate distinct but complementary metabolic effects. FXR is predominantly expressed in the liver and intestine and regulates bile acid synthesis through the FXR–FGF15/19 signaling axis [71,72]. Activation of FXR upregulates SHP and inhibits cholesterol 7α-hydroxylase (CYP7A1) and sterol 12α-hydroxylase (CYP8B1) to inhibit de novo bile acid synthesis; it also regulates the expression of apical sodium-dependent bile acid transporter (ASBT), sodium taurocholate co-transporting polypeptide (NTCP), and bile salt export pump (BSEP), balancing bile acid uptake, excretion, and enterohepatic circulation [73]. TGR5 is widely expressed in the intestine, brown adipose tissue, and skeletal muscle, where it increases intracellular cAMP levels, thereby activating AMPK via EPAC-dependent signaling pathways and GLP-1-mediated endocrine signaling [71,74]. These downstream signaling events are associated with enhanced GLP-1 secretion and increased energy expenditure [71,74], including brown adipose tissue thermogenesis, collectively contributing to glucose homeostasis and systemic energy balance [71,75].

Multiple animal studies have demonstrated that gut microbiota contribute substantially to bile acid homeostasis. Antibiotic administration markedly reduces gut microbial diversity and induces persistent disturbances in bile acid metabolism, highlighting the importance of an intact gut microbiota for maintaining bile acid homeostasis [76]. Furthermore, bile acid dysregulation exhibits distinct features under different metabolic conditions. In experimental models of T2DM, metabolic alterations are characterized by substantial remodeling of bile acid composition, including reduced levels of taurine-conjugated BAs and increased concentrations of secondary BAs such as DCA [77]. In contrast, HFD-induced obesity is primarily associated with disruption of the circulating bile acid pool, characterized by reduced hepatic and serum bile acid synthesis and increased fecal bile acid excretion, which has been associated with impaired ileal bile acid reabsorption [78].

By reshaping bile acid composition and modulating FXR and TGR5 signaling, microbiota-associated bile acid metabolites represent important regulatory intermediates linking dietary factors, microbial activity, and host metabolic homeostasis [8,10]. However, despite substantial mechanistic advances, evidence directly connecting microbiota-associated bile acid alterations with FXR/TGR5 signaling activation and metabolic improvement in large human cohorts remains limited [9]. Further clinical studies are needed to clarify the translational relevance of microbiota–bile acid signaling in MetS.

### 3.3. Trimethylamine N-Oxide

TMAO is a microbiota-associated metabolite generated through hepatic flavin-containing monooxygenase 3 (FMO3)-mediated oxidation of trimethylamine (TMA), which is produced by gut microbial metabolism of dietary substrates such as choline, L-carnitine, and betaine [79]. Increasing evidence suggests that elevated TMAO levels are associated with metabolic dysfunction, chronic inflammation, and cardiometabolic complications [79].

Several mechanisms have been proposed to explain the metabolic effects of TMAO. Experimental studies indicate that elevated TMAO may impair intestinal barrier integrity [80], promote metabolic endotoxemia, and activate inflammatory signaling pathways, including the toll-like receptor 4 (TLR4)/cluster of differentiation 14 (CD14)/NF-κB signaling axis and the NLRP3 inflammasome [80,81,82]. These pathways promote the production of pro-inflammatory cytokines such as interleukin-1β (IL-1β) and interleukin-18 (IL-18), thereby reinforcing inflammatory responses and contributing to chronic low-grade inflammation [81,82]. Overall, these inflammatory events converge to impair insulin signaling, contributing to systemic IR [83]. Furthermore, TMAO has been implicated in vascular dysfunction through potentiation of angiotensin II-mediated vasoconstrictive responses [84].

Emerging evidence suggests that TMAO may interact with host metabolic regulation through pathways involving bile acid–FXR signaling and immune cell metabolic reprogramming [85,86]. Moreover, circulating TMAO concentrations are influenced not only by microbial TMA production but also by host clearance capacity, suggesting that systemic TMAO levels reflect both microbial activity and host physiological processes [87].

The metabolic effects of TMAO appear to be highly context-dependent. In obese individuals with subclinical atherosclerosis, circulating TMAO concentrations are frequently elevated and positively associated with carotid intima–media thickness, IR, and central obesity [88]. However, studies in patients with early-stage MetS without overt cardiovascular disease or type 2 diabetes have reported no significant elevation in TMAO levels and no clear association with cardiometabolic risk [89].

In these individuals, gut microbial alterations are primarily characterized by enrichment of pro-inflammatory taxa and depletion of butyrate-producing bacteria, whereas taxa with known TMA-producing capacity, including members of the genera *Clostridium*, *Desulfovibrio*, and *Anaerococcus*, are not consistently increased [90,91,92]. These findings raise the possibility that inflammatory dysbiosis and TMAO-related metabolic alterations may emerge at different stages of disease progression.

Collectively, current evidence suggests that TMAO may serve as a biomarker and a potential modifier of cardiometabolic complications. However, the extent to which microbiota-associated alterations in TMAO metabolism influence MetS progression remains incompletely defined, and further longitudinal and mechanistic studies are required to clarify its stage-specific role in disease progression.

### 3.4. Branched-Chain Amino Acids

BCAAs, including leucine, isoleucine, and valine, have emerged as metabolic biomarkers associated with obesity, IR, and T2DM [93]. Numerous studies have reported elevated circulating BCAA levels in individuals with metabolic disorders, suggesting that disrupted BCAA metabolism is closely associated with metabolic dysfunction in MetS-related conditions [18].

In obesity and insulin-resistant states, excess lipid accumulation and chronic low-grade inflammation suppress the activity of the branched-chain α-keto acid dehydrogenase complex (BCKDH), the rate-limiting enzyme responsible for BCAA catabolism [94]. Impaired BCAA degradation may contribute to the accumulation of BCAAs and their intermediate metabolites in peripheral tissues and circulation [93,95]. Among circulating BCAAs, leucine is considered the most potent activator of the mechanistic target of rapamycin complex 1 (mTORC1) nutrient-sensing pathway, whereas isoleucine and valine exhibit weaker effects [96,97]. When all three accumulate concurrently, combined BCAA overload further enhances mTORC1 signaling [18].

Sustained activation of this nutrient-sensing pathway extends beyond translational control and impinges on cellular quality-control mechanisms, particularly autophagy and mitochondrial homeostasis [18]. Experimental studies indicate that excess BCAAs can activate mTORC1 signaling, thereby inhibiting ULK1-mediated autophagy through antagonism of AMPK–ULK1 signaling [18,98]. The resulting impairment of mitophagy leads to mitochondrial dysfunction and excessive ROS accumulation [99]. Increased oxidative stress suppresses SIRT3 expression, and reduced SIRT3 further downregulates PP2Cm expression, increases acetylation, and decreases activity of the BCKDH complex [100,101], potentially impairing BCAA degradation and creating a metabolic feedback loop that may further exacerbate metabolic imbalance [18]. Additionally, Chronic activation of mTORC1 by elevated BCAAs, particularly leucine, interferes with insulin signaling cascades and contributes to systemic dysregulation of glucose metabolism [102].

Beyond host metabolic phenotypes, gut microbiota represents one potential contributor to circulating BCAA variation [103]. Certain microbial taxa reported in metabolic disorders, including some members of the genus *Prevotella* and the family *Enterobacteriaceae*, have been suggested to possess BCAA biosynthesis potential and may contribute to variations in circulating BCAA profiles in the context of metabolic disorders [104]. Clinical studies have reported enrichment of BCAA-producing bacteria in some T2DM cohorts, and their abundance has been associated with fasting glucose levels and IR [104]. Experimental studies further indicate that colonization with BCAA-producing bacterial strains can impair glucose metabolism in mice, supporting a potential contribution of microbiota-associated BCAA metabolism to metabolic dysfunction [104].

Collectively, current evidence suggests that dysregulated BCAA metabolism represents an important metabolic pathway linking gut dysbiosis with IR and metabolic dysfunction [10,104]. In MetS, elevated circulating BCAA levels have been largely attributed to excessive dietary availability and impaired host BCAA catabolism, particularly reduced BCKDH-mediated oxidation [10]. However, emerging evidence suggests that gut microbial BCAA metabolism may further contribute to altered BCAA homeostasis by modulating intestinal BCAA availability and host metabolic responses [10,104]. Understanding the mechanisms underlying microbiota-associated BCAA regulation may facilitate the development of adjunctive microbiota-based approaches for MetS management.

### 3.5. Lipopolysaccharide

Chronic low-grade inflammation is widely recognized as a key contributor to the development of MetS [105]. Under physiological conditions, the intestinal barrier effectively prevents luminal microorganisms and microbial products from entering the systemic circulation. However, gut dysbiosis in MetS is frequently accompanied by impairment of the intestinal mucus layer and reduced expression of tight junction proteins [9,106]. These alterations increase intestinal permeability, resulting in a leaky gut phenotype that facilitates the translocation of bacterial products into the bloodstream [107].

Among these microbial products, LPS, a major component of the outer membrane of Gram-negative bacteria, plays a central role in metabolic endotoxemia [108]. Alterations in LPS-associated Gram-negative taxa, particularly members of the *Enterobacteriaceae* family and *Desulfovibrio*, may contribute to increased intestinal LPS burden and systemic exposure in metabolic disorders [105]. Elevated circulating LPS levels promote further disruption of intestinal barrier integrity and contribute to persistent systemic inflammation [108,109]. Mechanistically, LPS activates the TLR4 and CD14 signaling complex on immune cells [110], including macrophages, triggering downstream inflammatory pathways such as NF-κB [109]. This process stimulates the production of pro-inflammatory cytokines, including tumour necrosis factor-α (TNF-α), interleukin-6 (IL-6), pro-IL-1β and pro-IL-18, and activates the NLRP3 inflammasome for the maturation of IL-1β and IL-18 [108,109,110].

These inflammatory mediators converge on host metabolic regulation through multiple intersecting pathways. TNF-α and other pro-inflammatory cytokines activate stress kinases, including JNK and IKKβ, which impair insulin signaling by inducing inhibitory serine phosphorylation of insulin receptor substrate-1 (IRS-1) [111,112]. This reduces downstream PI3K/Akt signaling and impairs GLUT4 translocation and glucose uptake in skeletal muscle and adipose tissue, thereby promoting systemic insulin resistance [113]. Meanwhile, persistent inflammatory signaling and LPS-induced oxidative stress responses disrupt hepatic lipid metabolism by impairing AMPK activity and fatty acid oxidation [114], while promoting SREBP-1c-mediated lipogenic programs, thereby contributing to hepatic lipid accumulation and dyslipidemia [115]. Findings from experimental models suggest that gut microbiota disturbance may contribute to altered hepatic lipid metabolism, partly via dampened AMPK signaling cascades [116,117]. In addition, LPS-induced intestinal inflammatory responses may activate the JAK2/STAT3 signaling axis, potentially involving enhanced O-GlcNAcylation of STAT3 in intestinal epithelial cells, thereby contributing to intestinal inflammatory injury and barrier dysfunction [118]. Beyond intestinal barrier disruption, inflammation-associated oxidative stress impairs mitochondrial function and further amplifies metabolic dysfunction through a vicious cycle involving reactive oxygen species generation, inflammatory activation, and impaired energy metabolism [119,120]. LPS recognition through the CD14/TLR4 pathway links microbial inflammatory signals with host metabolic responses. In mice, chronic low-dose LPS induces obesity, glucose intolerance, dyslipidemia, and systemic inflammation in mice, whereas TLR4 deficiency confers protection against HFD-induced metabolic dysfunction [16]. Moreover, restoration of gut barrier integrity reduces circulating LPS and inflammation [9,121], and germ-free or microbiota-manipulated models support a microbiota–LPS–metabolism axis [16,121].

These experimental observations are further supported by evidence from human studies. In humans, circulating LPS and LBP are elevated in obesity, MetS, and type 2 diabetes and correlate with metabolic dysfunction and inflammatory markers [9]. Prospective cohort studies further show that elevated LBP predicts incident MetS [9]. Moreover, dietary interventions indicate that HFD has been associated with increased LPS levels and IR, whereas high-fiber diets have been reported to reduce systemic LPS and inflammation [9,121].

Collectively, these findings suggest that LPS represents an important microbiota-associated mediator linking gut microbial alterations to host inflammatory activation and metabolic dysfunction.

### 3.6. Crosstalk Among Microbiota-Associated Mediators

**Figure 2:**
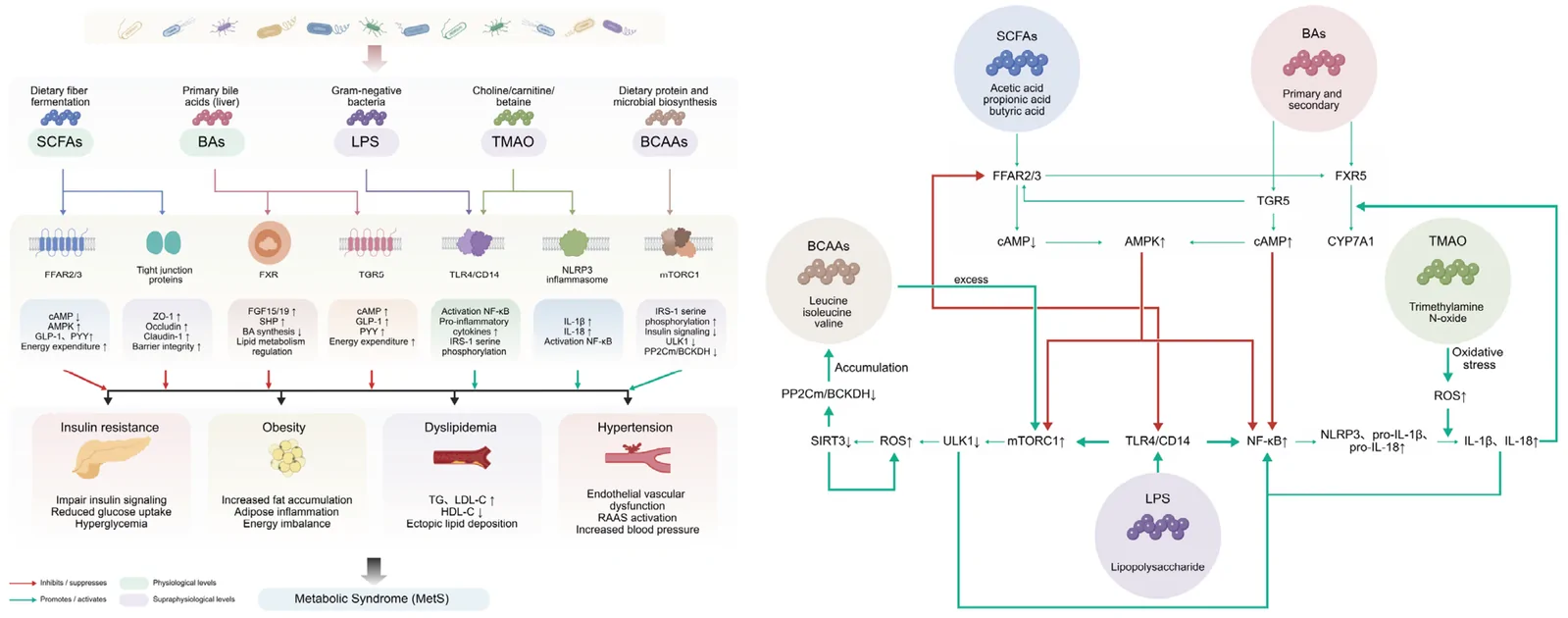
Integrated gut microbiota-associated mediator signaling network in MetS. This schematic illustrates core gut microbiota-associated mediators, which fall into three functional subgroups: metabolic regulators (SCFAs, BAs), inflammatory amplifiers (LPS, TMAO), and nutrient-sensing mediators (BCAAs). Beneficial signaling cascades mediated by SCFAs and BAs (FFAR2/3–AMPK, FXR/TGR5) may be impaired in MetS, while pathogenic TLR4/NF-κB/NLRP3 and mTORC1 signaling triggered by LPS, TMAO, and BCAAs may be enhanced. Crosstalk among these mediators may constitute an interconnected regulatory network that influences intestinal barrier integrity, inflammatory responses, and metabolic homeostasis. Alterations in this network have been associated with metabolic disturbances, including IR, obesity, dyslipidemia, and hypertension. Created with BioGDP.com [27].

Gut microbiota-associated mediators collectively form an interconnected metabolic signaling network that may influence host energy metabolism, immune responses, and systemic homeostasis [9]. Rather than acting independently, these mediators interact within an integrated multi-layered system that links gut microbiota-associated metabolic signals with host signaling pathways [9]. In MetS, alterations in this network are associated with metabolic dysfunction, chronic low-grade inflammation, and impaired intestinal barrier integrity (Figure 2).

#### 3.6.1. SCFA–BA Signaling Crosstalk Axis

SCFAs and BAs represent important microbiota-associated mediators that interact with host metabolic signaling pathways. SCFAs activate FFAR2/3 signaling, modulating intracellular cAMP and Ca^2+^ signaling, which may influence FXR transcriptional activity and bile acid metabolism [122]. Conversely, BAs activate TGR5 signaling, leading to increased cAMP production and downstream PKA–CREB activation, which may influence FFAR2/3 expression and enhance cellular responsiveness to SCFAs [123]. This bidirectional interaction may contribute to the coordination of GPCR- and nuclear receptor-mediated signaling pathways involved in host–microbe metabolic communication [122,123].

#### 3.6.2. SCFA–LPS Gut Barrier–Inflammation Axis

SCFAs have been associated with maintaining intestinal barrier integrity and regulation of inflammatory responses. Through FFAR2/3 activation and AMPK signaling, SCFAs may inhibit NF-κB-mediated inflammatory responses and reinforce epithelial barrier function, thereby potentially limiting systemic exposure to LPS [92]. In contrast, reduced intestinal SCFA levels have been associated with increased intestinal permeability and enhanced LPS translocation, resulting in activation of the TLR4/CD14–NF-κB signaling axis and amplification of systemic inflammation [124]. Notably, inflammatory signaling induced by LPS further suppresses SCFA receptor expression, reducing host sensitivity to SCFA-mediated anti-inflammatory effects and potentially reinforcing a self-amplifying pro-inflammatory feedback loop [92].

#### 3.6.3. SCFA–BCAA Metabolic Stress Regulation Axis

SCFAs and BCAAs form interconnected metabolic regulators that converge on mitochondrial function and BCAA catabolism rather than exerting direct reciprocal regulation. SCFAs may activate FFAR2/3–AMPK signaling to enhance mitochondrial metabolic activity, which may indirectly influence BCAA catabolism and metabolic homeostasis [125]. In contrast, excessive BCAAs disrupt cellular energy sensing through mTORC1–AMPK imbalance and reduced BCAA catabolic capacity [18]. Together, these opposing influences converge on mitochondrial metabolic flexibility and contribute to the maintenance or disruption of systemic metabolic balance [18].

#### 3.6.4. LPS–TMAO Inflammatory Amplification Loop

LPS and TMAO represent inflammatory-associated mediators within this metabolite network. Increased intestinal permeability facilitates LPS translocation, which activates the TLR4/CD14–NF-κB axis and promotes systemic inflammatory responses [9,121]. Concurrently, TMAO has been associated with oxidative stress and activation of NLRP3 inflammasome-associated inflammatory pathways, contributing to vascular and metabolic inflammation [45]. Importantly, both LPS and TMAO can enhance inflammatory responses through activation or amplification of NF-κB-associated signaling pathways. These inflammatory processes may further interact with intestinal barrier dysfunction, forming a sustained inflammatory feedback loop that exacerbates metabolic dysregulation [9,45,121].

#### 3.6.5. BAs–TMAO–BCAA Metabolic Interaction Axis

BAs further interact with amino acid and choline metabolism axes through microbiota–host signaling networks. Bile acid–FXR signaling may influence microbial composition and metabolic activity, thereby potentially affecting TMA production [10,45]. In parallel, elevated BCAAs activate mTORC1 signaling and may impair bile acid metabolic regulation through the FXR–FGF15/19 axis disruption [18,126]. Additionally, inflammatory and metabolic stress states further enhance TMAO- and BCAA-associated metabolic disturbances, thereby influencing systemic metabolic homeostasis [10].

#### 3.6.6. Integrated Network and System-Level Synthesis

Collectively, alterations in these mediators may represent important modulatory factors that exacerbate metabolic dysregulation and chronic low-grade inflammation in the context of MetS [10]. Within this system, SCFAs and BAs primarily function as homeostatic regulators that maintain epithelial integrity and coordinate host metabolic signaling through GPCR- and nuclear receptor-mediated pathways [71,92]. In contrast, LPS and TMAO act as pro-inflammatory amplifiers that destabilize immune–metabolic balance by promoting oxidative stress and inflammatory signaling cascades [45]. BCAAs occupy an intermediate layer linking nutrient sensing to mitochondrial function and metabolic stress responses through mTORC1- and AMPK-dependent pathways [18].

Importantly, these mediators are not functionally isolated but are organized into an interconnected regulatory network [9,10]. Lifestyle-related metabolic stressors, including excessive caloric intake and reduced physical activity, can influence gut microbial composition and microbiota-associated mediator profiles [8,9]. These changes may alter microbiota–mediator–host signaling interactions, potentially amplifying pre-existing metabolic disturbances, inflammatory responses, and intestinal barrier dysfunction associated with MetS [9,10].

Notably, modulating gut microbiota composition, associated mediator profiles, and host response pathways may represent a potential strategy to support metabolic homeostasis and complement existing interventions for MetS management [10,125].

### 3.7. Multi-Omics Analysis of Microbiota–Mediator–Host Interactions

In recent years, advances in multi-omics approaches and long-read sequencing technology have provided powerful tools for investigating complex microbiota–mediator–host signaling networks [127,128,129]. Although 16S rRNA sequencing has substantially improved our understanding of microbial community alterations associated with metabolic disorders, it cannot fully capture the functional and metabolic processes underlying microbiota–host communication [130]. Multi-omics approaches integrating metagenomics, metatranscriptomics, metabolomics, and proteomics provide complementary strategies to investigate these complex interactions by linking microbial functional potential, microbial activity, metabolite production, and host molecular responses [131].

Metagenomics provides a broad overview of the functional genetic potential of gut microbial communities beyond taxonomic classification [130,132]. This approach enables the identification of microbial pathways associated with the synthesis, degradation, or transformation of microbiota-associated mediators [9,132]. However, metagenomic profiles primarily reflect the functional capacity encoded within microbial genomes and cannot determine whether these pathways are actively expressed under specific physiological conditions [127,130].

Metatranscriptomics complements metagenomic analysis by capturing actively transcribed microbial genes and the transcriptional activity of microbial communities [132,133]. By distinguishing microbial functional potential from active gene expression profiles, metatranscriptomics enables the characterization of microbial transcriptional activity under metabolic disturbances, dietary interventions, or microbiota-targeted therapies [9,132]. Integration of metagenomic and metatranscriptomic data therefore helps distinguish microbial functions that are actively expressed from those that merely reflect differences in microbial abundance or genomic potential [9,132,133,134].

Among multi-omics approaches, metabolomics provides a critical functional layer linking microbial activity with host metabolic regulation [10,135]. Microbiota-associated mediators represent key molecular intermediates connecting microbial metabolism with host signaling pathways [135]. Metabolomic profiling enables direct measurement of these molecules and may reveal functional alterations that cannot be inferred solely from microbial composition [127]. In this context, metabolomics facilitates a shift from taxonomy-based interpretation toward function-oriented analysis of microbiota–host interactions.

Proteomics further expands network analysis by characterizing host molecular responses associated with microbiota-associated mediator signaling [130,131]. Alterations in proteins involved in inflammation, insulin signaling, lipid metabolism, and immune regulation may help identify host response pathways linked to microbial mediator activity [131]. Integrated analysis of metabolomic and proteomic data may further facilitate the exploration of host signaling pathways associated with microbiota-associated mediators [127,131], including those involving SCFA–FFAR2/3, bile acid–FXR/TGR5, and tryptophan-derived metabolite–AhR signaling.

The integration of multi-omics datasets using computational approaches, including network analysis and pathway modeling, facilitates the development of integrative frameworks for understanding microbiota–host interactions [136]. By combining microbial functional profiles, metabolite signatures, and host molecular responses, multi-omics approaches can generate mechanistic hypotheses and explore potential microbiota–mediator–host regulatory pathways [137,138]. Furthermore, longitudinal multi-omics studies integrated with targeted interventions and experimental validation may help clarify the biological mechanisms underlying microbiota–mediator–host signaling.

## 4. Gut Microbiota-Targeted Therapeutic Strategies in MetS

Within the microbiota–mediator–host signaling network involved in metabolic homeostasis, environmental and behavioral inputs exert upstream influences on gut microbial functions [8,10]. Lifestyle factors, particularly dietary patterns and physical activity levels, represent major determinants of metabolic dysfunction and can also influence gut microbial composition and function [8,139]. Increasing evidence indicates that microbiota-associated mediators act as important intermediates between lifestyle factors and host metabolic regulation [125,139]. Accordingly, interventions targeting the gut microbiota and associated mediators have gained increasing attention as potential strategies for modulating metabolic homeostasis [140].

### 4.1. Probiotics, Prebiotics, Synbiotics

Randomized controlled trials (RCTs) and meta-analyses suggest that probiotics, prebiotics, and synbiotics may exert modest beneficial effects on several metabolic parameters in individuals with MetS, including body weight, waist circumference, serum triglycerides, and fasting blood glucose [141]. However, the clinical efficacy of these interventions remains heterogeneous and is influenced by host characteristics, dietary patterns, baseline gut microbiota composition, intervention protocols, and microbial strains (Table 2). For example, meta-analyses indicate that metabolic improvements may be pronounced in Asian populations, individuals younger than 50 years, and interventions lasting less than 12 weeks [141]. Additional systematic reviews suggest that multi-strain probiotics and substrate-matched synbiotics may improve glycemic control, including reductions in glycated hemoglobin and improvements in IR [142]. These clinical outcomes have been associated with alterations in gut microbiota-associated mediators, including increased levels of butyric acid and ursodeoxycholic acid (UDCA) [142], suggesting that the metabolic effects of microecological interventions may depend largely on functional remodeling of microbial activity rather than microbial supplementation alone.

Mechanistic studies have further provided insights into how probiotics, prebiotics, and synbiotics may influence host metabolism through the microbiota–mediator axis (Table 3). Rather than simply increasing the abundance of beneficial bacteria, these interventions may alter microbial functions and metabolic outputs, thereby influencing host signaling pathways. For instance, although *Lactobacillus reuteri* ZJ617 cannot synthesize spermidine by itself, it may promote the synthesis of spermidine by intestinal polyamine-producing microorganisms by providing the precursor S-adenosylmethionine (SAM). This microbial cross-feeding mechanism may promote white adipose tissue browning and improved metabolic outcomes in HFD-fed mice [143]. Similarly, the dietary fiber prebiotics polydextrose (PDX) can remodel gut microbiota communities and activate the intestinal bile acid receptor FXR signaling, which further suppresses the expression of lipid absorption-related genes Dgat1 and Cd36, contributing to hypolipidemic effects [144].

Beyond conventional microbial supplementation, increasing attention has been directed toward targeting gut microbiota-associated mediators and their host signaling pathways. For instance, aromatic amino acid metabolites, including 4-hydroxyphenylacetic acid (4HPAA) and its analogues 3HPP and 4HPP, have shown anti-obesity potential by suppressing intestinal inflammation and lipid absorption [145]. Similarly, indolepropionic acid (IPA), a microbial tryptophan metabolite, has been implicated in regulating immune homeostasis and renal sodium metabolism, suggesting a potential role in salt-sensitive hypertension [146]. Recent studies have highlighted the potential role of microbiota-derived indole metabolism in activating aryl hydrocarbon receptor (AhR) signaling, which may serve as a molecular intermediate linking microbial metabolism, intestinal barrier integrity, and host metabolic regulation [147]. Under pathological states such as MetS, the microbiota’s ability to produce AhR agonists is impaired. This may result in reduced AhR activation, which is associated with impaired intestinal barrier integrity, altered GLP-1 release, and metabolic dysfunction [148]. Preclinical evidence suggests that supplementation with AhR agonists or AhR-producing probiotics may effectively improve these pathways [148], providing a conceptual framework for developing microbiota–mediator–host signaling axes.

Despite their potential metabolic benefits, the clinical translation of probiotics, prebiotics, and synbiotics remains limited by several unresolved challenges. The metabolic effects of these interventions are highly heterogeneous and are influenced by microbial strains, intervention protocols, host characteristics, dietary patterns during the intervention period, and baseline gut microbiota composition [149]. In particular, the benefits of probiotics are often strain-specific, and the colonization and functional persistence of administered microorganisms in the recipient gut remain variable [21]. Similarly, the efficacy of prebiotics depends largely on whether the resident gut microbiota can effectively utilize these substrates and produce beneficial metabolites [150]. Importantly, clinical improvements observed in some studies are often achieved alongside dietary modification and increased physical activity, indicating that microbiota-targeted interventions are most likely to provide benefits when integrated with established lifestyle management [151]. Although mechanistic studies have suggested potential pathways involving microbiota-associated mediators and host signaling regulation, the causal links between these mechanisms and clinical outcomes remain incompletely established in humans. Therefore, a more precise understanding of host–microbiota interactions and microbial functional responses is required to improve the clinical translation of probiotics, prebiotics, and synbiotics in metabolic disorders.

**Table 2:** Clinical evidence and metabolic outcomes of probiotics and synbiotics in metabolic disorders.

Type	Supplements	Population	Dosage Regimen	Outcomes	Mechanism	Ref.
Probiotics	LRC UALp-05 B420	American MetS	6 × 10^9^ CFU/d, 4 × 10^9^ CFU/d, 1 × 10^10^ CFU/d, oral, 10 weeks	Responder: TG, DBP ↓ Non-responder: FBG, INS, HOMA-IR ↑	Modulates bile acid biotransformation and reduces metabolic endotoxemia.	[152]
	*L. acidophilus* *L. plantarum* *B. lactis* *S. boulardii*	Greek T2DM	1.75 × 10^9^ CFU/d, 0.5 × 10^9^ CFU/d, 1.75 × 10^9^ CFU/d, 1.5 × 10^9^ CFU/d, oral, 24 weeks	HbA1c, FBG, TC, WC ↓	Probiotics restore gut barrier, upregulate GLUT4 and modulate insulin synthesis.	[153]
	HY7601 KY1032	South Korean Obesity	5 × 10^9^ CFU/d, 5 × 10^9^ CFU/d, oral, 12 weeks	BW, WC, BMI, BFM, VF ↓	Modulates gut microbiota, improves adipokine secretion, alleviates leptin resistance, and ameliorates obesity.	[154]
	AKK-WST01	Chinese Obesity+ T2DM	3–15 × 10^10^ CFU/d, oral, 12 weeks	LBL: BW, BMI, BFM, VF, HbA1c, FBG, BP, LDL-C ↓ HBL: no significant improvement	Endogenous *Akkermansia* occupies niche to inhibit AKK-WST01 colonization. Colonized strain repairs gut barrier and boosts energy expenditure and GLP-1 secretion.	[155]
Synbiotics	NCFM HN019 PDX	Chinese Obesity	1 × 10^10^ CFU/d, 1 × 10^10^ CFU/d, 3.4 g/d, oral, 8 weeks	No significant improvement	Restore the intestinal barrier, modulate gut microbiota, produce acetate, promote gut satiety hormone secretion, enhance insulin sensitivity.	[156]

Abbreviations: LRC = *Limosilactobacillus reuteri* NCIMB 30242; UALp-05 = *Lactiplantibacillus plantarum* UALp-05™; B420 = *Bifidobacterium animalis* subsp. lactis B420™; *L. acidophilus* = *Lactobacillus acidophilus*; *L. plantarum* = *Lactobacillus plantarum*; *B. lactis* = *Bifidobacterium lactis*; *S. boulardii* = *Saccharomyces boulardii*; HY7601 = *Lactobacillus curvatus* HY7601; KY1032 = *Lactobacillus plantarum* KY1032; AKK-WST01 = *Akkermansia muciniphila* WST01; NCFM = *Lactobacillus acidophilus* NCFM; HN019 = *Bifidobacterium lactis* HN019; TG = Triglycerides; TC = Total Cholesterol; LDL-C = low-density lipoprotein cholesterol; DBP = Diastolic blood pressure; BP = Blood pressure; FBG = Fasting Blood Glucose; HbA1c = Glycated Hemoglobin A1c; INS = Insulin; HOMA-IR = Insulin resistance index; WC = Waist circumference; BW = Body weight; BMI = Body Mass Index; BFM = Body Fat Mass; VF = Visceral Fat; GLUT4 = Glucose Transporter Type 4; GLP-1 = Glucagon-like peptide-1; PDX = Polydextrose; LBL = Low baseline level; HBL = High baseline level.

**Table 3:** Preclinical evidence and mechanistic insights into the metabolic effects of probiotics, prebiotics, and synbiotics.

Type	Supplements	Disease	Dosage Regimen	Outcomes	Mechanism	Ref.
Probiotics	EF-1	Obesity	2 × 10^9^ CFU/d, gavage, 16 weeks	Reduced body weight gain, decreased adiposity, and improved lipid metabolic profile.	Probiotics restore the intestinal barrier, enhance BSH-mediated bile salt hydrolysis, inhibit glucosidase activity, and improve glycolipid metabolism.	[157]
	HM108	Obesity	LD: 2.5 × 10^8^ CFU/d, MD: 5 × 10^8^ CFU/d, HD: 1.5 × 10^9^ CFU/d, gavage, 6 weeks	Reduced body weight gain, decreased adiposity, and improved lipid metabolic profile.	Modulates gut microbiota, suppresses JAK-STAT signaling activation, and improves systemic metabolic and inflammatory status.	[158]
	LA-1	Obesity	2 × 10^9^ CFU/d, gavage, 16 weeks	Reduced body weight gain, decreased adiposity, and improved lipid metabolic profile.	Modulates gut microbiota composition, enhances SCFA-producing bacteria, suppresses inflammation, and improves host lipid metabolism.	[159]
	*F. prausnitzii*	T2DM	1 × 10^8^ CFU/tiw gavage, 5 weeks	Improved insulin sensitivity, improved lipid metabolic profile, and reduced inflammatory status.	Enhances butyrate production, inhibits NF-κB signaling, and reduces metabolic inflammation.	[35]
	AKK-WST01	Obesity	2 × 10^8^ CFU, 6 times weekly, gavage, 5 weeks	Improved glucose tolerance, reduced body weight gain, and enhanced insulin sensitivity.	Promotes mucin layer remodeling and tight junction protein expression, strengthens intestinal barrier integrity, and suppresses LPS-induced inflammatory signaling, leading to improved metabolic homeostasis.	[155]
Prebiotics	CGP	Obesity	400 mg/kg·d gavage, 8 weeks	Improved hepatic steatosis, reduced lipid accumulation, and alleviated liver injury.	Modulates gut microbiota, increases SBAs to activate TGR5 pathway and enhance thermogenesis, thereby alleviating obesity.	[160]
	GOS SA	Obesity	LD: 1.7 g/kg·d, HD: 8.5 g/kg·d, LD: 50 mg/kg·d, HD: 250 mg/kg·d, gavage, 6 weeks	Ameliorated metabolic disorders, reduced body weight gain, and improved lipid and glucose metabolism.	Restore the intestinal barrier, reduce endotoxin leakage, suppress inflammation, upregulate GLUT4, inhibit lipid absorption, and synergistically improve metabolic disorders.	[161]
	BDF	T2DM	10% BDF in feed, free access to food, 8 weeks	Improved glucose homeostasis, enhanced insulin sensitivity, and alleviated T2DM metabolic dysfunction.	Modulates gut microbiota and bile acid metabolism, activates FXR/TGR5 signaling, and restores IRS-1/PI3K/AKT insulin pathway to improve glucose metabolism.	[162]
	Inulin	Hypertension	Inulin-substituted AIN-76A diet, free access to food, 5 weeks	Attenuated salt-sensitive hypertension and reduced renal damage.	Modulates gut microbiota and SCFA signaling (propionate), reduces inflammation, and improves vascular and renal function.	[163]
	Arabinoxylan	Obesity	200 mg/kg·d, gavage, 4 weeks	Reduced body weight gain, decreased adiposity, and improved lipid metabolic profile.	Modulates gut microbiota, boosts DCA production to activate TGR5 and stimulates GLP-1 secretion, thus improving glycolipid metabolism.	[164]
Synbiotics	LPm77 Inulin	T2DM	1 × 10^9^ CFU/d, gavage, 10% inulin-supplemented diet, free access to food, 7 weeks	Improved glucose homeostasis and insulin sensitivity and relieved systemic inflammation.	Modulates gut microbiota and enhances TUDCA metabolism, regulating the gut–liver axis and improving glucose metabolism.	[165]
	*L. acidophilus* *B. infantis* KGMO	Obesity	1.5 × 10^9^ CFU/d, 1.5 × 10^9^ CFU/d, 2 g/kg·d, oral gavage, 12 weeks	Reduced body weight gain, decreased adiposity, and improved lipid metabolic profile.	Modulates gut microbiota and lipid metabolism, inhibits hepatic TLR4/NF-κB signaling, and reduces inflammation and obesity.	[166]
	K56+ XOS/GOS/PG	Obesity	3 × 10^8^ CFU/mL, 4 g/L, ferment for 24 h	Improved intestinal microecology and metabolic status.	Selectively promote beneficial bacteria, optimize SCFA profiles, and improve gut dysbiosis.	[167]

Abbreviations: EF-1 = *Enterococcus faecalis* EF-1; HM108 = *Limosilactobacillus reuteri* HM108; LA-1 = *Ligilactobacillus animalis* LA-1; *F. prausnitzii* = *Faecalibacterium prausnitzii*; AKK-WST01 = *Akkermansia muciniphila* WST01; LPm77 = *Lactiplantibacillus plantarum* LPm77; *L. acidophilus* = *Lactobacillus acidophilus*; *B. infantis* = *Bifidobacterium infantis*; K56 = *Lacticaseibacillus paracasei* K56; F/B = Firmicutes/Bacteroidetes; SCFAs = Short-chain fatty acids; SBAs = Secondary bile acids; DCA = Deoxycholic acid; TUDCA = Tauroursodeoxycholic acid; BSH = Bile Salt Hydrolase; GLUT4 = Glucose Transporter Type 4; GLP-1 = Glucagon-like peptide-1; TGR5 = Takeda G protein-coupled receptor 5; CGP = Cordyceps guangdongensis polysaccharides; GOS = Galactooligosaccharides; SA = Sodium alginate; BDF = Total dietary fiber of tartary buckwheat; KGMO = Konjac glucomannan; XOS = Xylooligosaccharide; PG = Polyglucose; LD = Low dose; MD = Medium dose; HD = High dose.

### 4.2. Fecal Microbiota Transplantation

FMT represents an approach for directly transferring complex microbial communities and has attracted increasing research interest. FMT can lead to marked changes in recipient microbial composition and functional profiles. For recurrent *Clostridioides difficile* infection (CDI), joint guidelines from the British Society of Gastroenterology (BSG) and the Healthcare Infection Society (HIS) recommend FMT as an established treatment option following adequate antibiotic pretreatment [168]. Its single-session cure rate reaches 80–90%, which is markedly higher than that of vancomycin [169].

In contrast to CDI, the clinical efficacy of FMT in metabolic diseases remains uncertain and highly heterogeneous. For example, RCTs in metabolic dysfunction-associated steatotic liver disease (MASLD) have reported limited efficacy in reducing hepatic fat accumulation [170], suggesting disease-specific variability in therapeutic response. Van der Vossen et al. published solid evidence supporting this mechanism. In patients with MetS, higher colonization of donor strains correlated with greater reductions in diastolic blood pressure [171]. This regulatory effect is mediated by *Collinsella aerofaciens* and *Fusocatenibacter saccharivorans*, which can lower circulating 2-oxoarginine [171]. These findings indicate that the potential importance of donor strain engraftment, stable microbial colonization, and functional metabolic integration in determining FMT responses, although causal relationships remain to be established.

Despite its potential advantages, several challenges limit the application of FMT in chronic metabolic diseases. These include highly invasive delivery approaches (colonoscopy, nasojejunal intubation, and rectal enema) and low patient compliance [172,173]. To improve feasibility and standardization, efforts have focused on developing alternative delivery approaches, particularly oral FMT formulations. Srinivas Kamath et al. comprehensively reviewed transformation strategies for oral FMT, including freeze-drying for capsule preparation, enteric coating to protect viable microbes, and sensory optimization [174]. Preliminary clinical studies suggest that these formulations are feasible and may retain short-term efficacy in hypertension and MASLD [175,176].

Beyond these advances in delivery optimization, several additional challenges continue to limit the clinical translation of FMT in MetS. Therapeutic responses remain highly heterogeneous, likely reflecting differences in donor microbiota composition, recipient baseline microbial ecology, and host metabolic characteristics [177]. More specifically, the extent and durability of donor microbial engraftment may contribute to variability in treatment outcomes, although the underlying determinants remain incompletely understood [178,179]. In addition, the lack of standardized frameworks for donor selection, microbial characterization, dosing strategies, and long-term monitoring represents a major barrier to clinical implementation [177]. Beyond issues of standardization, several safety concerns and ecological uncertainties remain to be addressed during the clinical translation of FMT. These include potential pathogen transmission, transfer of antimicrobial resistance genes, spatial mismatch between donor microorganisms and recipient gut niches, unintended perturbation of recipient microbial ecosystems, and poorly characterized long-term consequences for host immune and metabolic regulation [177,180].

Overall, FMT represents an evolving microbiota-based intervention that is transitioning from empirical microbiota replacement toward more precise approaches guided by donor–recipient compatibility and functional metabolic activity. However, the role of FMT as an adjunctive intervention in MetS management remains to be established, requiring further clinical validation to define its efficacy, safety, and optimal integration with established lifestyle and therapeutic approaches.

### 4.3. Live Biotherapeutic Products

LBPs represent an emerging strategy for advancing precision microbiome-based intervention. As defined by the FDA guidelines issued in 2016, LBPs are biological products containing viable microorganisms for the prevention, treatment, or cure of human diseases, and they are classified separately from vaccines [181]. Benefiting from the rapid development of synthetic biology and microbiology [182], LBPs are grouped into three major types: single-strain preparations, defined microbial consortia, and genetically engineered bacteria, with great potential for the treatment of metabolic diseases, cancers, and infectious diseases [183].

Recent clinical advances highlight the transition of LBPs from empirical microbial supplementation to rationally designed, mechanism-driven therapeutics. RBX2660, a stool-associated microbial product, has indicated sustained efficacy in recurrent CDI [184], while SER-109, composed of purified Firmicutes spores, significantly reduces CDI recurrence rates in phase III clinical trials [185]. VE303, an eight-strain defined microbial consortium, further has demonstrated dose-dependent therapeutic efficacy [186], supporting the feasibility of rationally designed microbial communities.

Beyond CDI, LBPs are being investigated in metabolic and systemic diseases. Engineered strains such as SYNB1618, which degrade phenylalanine for the treatment of phenylketonuria [187], represent a microbial cell factory approach for treating metabolic disorders. Similarly, candidate LBPs, including *Akkermansia muciniphila*, have shown potential for improving insulin sensitivity and metabolic homeostasis [155]. These studies suggest that LBPs may enable the development of function-oriented microbiota therapeutics beyond conventional microbial supplementation.

Mechanistically, LBPs are designed to achieve targeted modulation of gut microbial functions and microbiota-associated mediator production, thereby influencing host metabolic and immune responses. Compared with conventional probiotics, LBPs aim to provide more defined microbial compositions and reproducible biological activities [188], thereby facilitating the investigation of specific microbiota–mediator–host signaling pathways. With continued advances in synthetic biology, genome editing, and targeted delivery systems, LBPs may complement existing microbiota-based interventions and contribute to the development of next-generation microbiome therapeutics.

Despite their rational design and potential for precision microbiome therapy, several unresolved issues remain before LBPs can be effectively applied in metabolic disorders. First, clinical evidence supporting the efficacy of LBPs in metabolic diseases remains limited. Although LBPs have demonstrated clinical benefits in recurrent CDI, most metabolic applications are still supported primarily by preclinical studies, and large-scale, long-term randomized controlled trials are required to establish their efficacy, safety, and optimal therapeutic strategies [188,189]. Second, maintaining stable and controllable microbial functions remains a major challenge. Although LBPs are designed for defined functions, ensuring consistent expression of intended functions remains difficult, particularly for engineered strains producing specific metabolites or therapeutic molecules [187]. For genetically engineered LBPs, additional biosafety considerations include genetic stability, reliable biocontainment, potential horizontal transfer of engineered genetic elements, and unintended environmental release after excretion [189]. Third, regulatory and manufacturing frameworks for LBPs remain under development, requiring further standardization of production processes, batch-to-batch consistency, functional characterization, and biosafety evaluation [189]. Therefore, despite their potential as precision microbiome therapeutics, LBPs remain an emerging platform, and further studies are needed to optimize functional stability, ensure safety, and establish effective regulatory frameworks.

## 5. New Progress of Gut Microbiota-Targeted Intervention Technologies

Recent advances in microbiome engineering are facilitating a transition from conventional microbial supplementation toward more dynamic, programmable, and precision-controlled therapeutic systems. These technological advances provide new opportunities to improve microbial design, delivery, and functional control, thereby facilitating the development of next-generation microbiome therapeutics.

In engineered bacteria–device interface systems, ingestible optoelectronic capsules have been explored to enable bidirectional communication with gut-resident engineered microbes [190]. These systems allow real-time sensing of intestinal biochemical cues and external modulation of engineered bacterial gene expression, providing a framework for dynamic monitoring and regulation of the gut microenvironment.

Biosensor-driven engineered bacterial systems built on environmental sensing circuits enable autonomous detection of disease-relevant microenvironments and controlled activation of therapeutic functions [191]. These systems provide a potential closed-loop strategy integrating targeted colonization, therapeutic delivery, and controlled clearance of engineered microbes.

In the field of microbiome-based delivery systems, Jinquan Li and colleagues have developed a non-invasive oral phage delivery platform (SA/HA/ES hydrogel microspheres) that selectively targets and lyses *Salmonella* [192]. Meanwhile, the prebiotic components in the microspheres promote the growth of beneficial bacteria, thereby enabling targeted modulation of gut microbial communities [192]. Nanozyme-modified probiotic delivery systems enhance targeted colonization and improve antioxidant capacity at inflammatory sites, addressing limitations associated with conventional probiotic therapies, including challenges in maintaining stability and achieving site-specific delivery [193].

Despite rapid progress in microbiome engineering technologies, the clinical translation of these approaches remains limited by challenges in safety validation, large-scale manufacturing, and regulatory standardization. Future development should focus on integrating programmable microbial systems with controllable delivery platforms to achieve clinically applicable precision microbiome therapeutics.

## 6. Discussion

Although accumulating evidence suggests that probiotics, prebiotics, and synbiotics may influence metabolic outcomes through microbiota-associated mechanisms, most available data remain largely correlative and lack definitive causal validation [194,195]. Current studies have not yet established complete mechanistic cascades linking specific microbial strains, microbial mediators, and host receptor signaling pathways [194,196]. Consequently, the molecular underpinnings of microbiota–mediator–host signaling axes remain incompletely defined [195,196]. Future investigations should integrate germ-free and humanized models, gene-editing approaches, multi-omics profiling, and well-designed human studies to move beyond association and establish causal relationships, focusing on core mediator–host signaling interactions including SCFAs–FFAR2/3, BAs–TGR5/FXR, and tryptophan metabolites–AhR signaling [92,194,196].

Despite their potential, multi-omics approaches still face several challenges. These include high-dimensional data integration, differences in analytical platforms and bioinformatic pipelines, limited longitudinal human studies, and insufficient experimental validation of multi-omics-derived hypotheses [137]. In addition, microbial functions and metabolite profiles are influenced by host genetics, diet, medication use, and environmental exposures, which complicate causal interpretation [52]. Therefore, future applications of multi-omics approaches will require integration with well-designed longitudinal cohorts and functional validation models to improve mechanistic understanding of microbiota–mediator–host signaling interactions in MetS.

Beyond unresolved mechanistic questions and technical limitations, substantial inter-individual heterogeneity acts as a major barrier to translational reproducibility in microbial intervention research [197,198]. Many trials are constrained by single-center designs, limited sample sizes, and short intervention periods, generating variable and generally modest therapeutic outcomes [197,198]. Even under standardized synbiotic regimens, clear discrepancies between responders and non-responders are widely observed, driven by differences in baseline gut microbiota, host metabolic phenotypes, habitual diet and environmental exposures [197,198,199,200]. Importantly, metabolic improvements reported in clinical studies are often achieved together with dietary modification and lifestyle optimization, making it difficult to determine the independent contribution of microbiota-targeted interventions [149]. Therefore, future studies should adopt precision approaches integrating host characteristics, baseline microbiome architecture, and microbial functional capacity to improve therapeutic predictability [197,198,199,200].

Of note, as a comprehensive whole-community microbial intervention distinct from conventional probiotic and synbiotic supplementation, FMT further highlights the translational challenges of gut microbiota modulation. As a whole-community microbial intervention involving bacteria, fungi, archaea, and viruses, FMT reflects the complexity of translating ecological manipulation into predictable clinical outcomes, particularly regarding microbial persistence, functional stability, and long-term host–microbiota interactions [173,201]. Although bacteriophages and other non-bacterial microorganisms may contribute to therapeutic effects, their functional roles remain insufficiently characterized [201,202].

Additionally, standardized operational frameworks for microbiota-based therapeutics are still absent, including unified dosing criteria, administration routes, and long-term follow-up schedules [203,204,205,206]. Critical safety risks, such as horizontal transmission of antibiotic resistance genes, persistent strain colonization, and potential tumorigenic hazards, remain insufficiently characterized and require systematic evaluation [203,204,205]. From a global translational perspective, inconsistent standardization and divergent regulatory rules across regions hinder broad clinical application and approval of microbial therapeutics [183,207,208]. Many microbial products are classified as dietary supplements rather than pharmaceuticals, escaping rigorous pharmaceutical supervision [183,207]. Ambiguous strain traceability, inconsistent functional claims, and ethical concerns surrounding genetically engineered live biotherapeutics further complicate clinical translation [183,207]. Recent international microbiota consortium consensus statements highlight the urgent demand for harmonized global regulatory frameworks governing probiotics, LBPs, and FMT [183,207,208]. Construction of universal strain repositories, unified clinical trial protocols, and multi-center real-world evidence cohorts will be essential to push this field forward [183,207,208].

Despite substantial inter-cohort variation in gut taxonomic composition across MetS populations, some microbial metabolic patterns and metabolic outputs may show greater consistency, suggesting potential functional convergence beyond taxonomic differences. This observation suggests the value of integrating microbial functional characteristics and microbiota-associated mediators with taxonomy analyses to provide deeper insights into microbiota–mediator–host communication in MetS [194,195]. Gut microbiota-associated mediators represent important molecular links between microbial activity and host signaling pathways and may provide insights into potential mechanisms involved in metabolic regulation and inform the development of targeted microbiota-based adjunctive strategies for MetS.

## 7. Conclusions

Metabolic syndrome is primarily driven by chronic positive energy balance, excessive caloric intake, reduced physical activity, and associated host metabolic stress. Within this context, gut microbiota-associated mediators may serve as important signaling intermediates linking lifestyle-related environmental factors, microbial functional activity, and host metabolic responses. The microbiota–mediator–host signaling network proposed in this review offers a conceptual framework for understanding how microbiota-associated metabolic signals may modulate intestinal barrier function, inflammatory responses, glucose homeostasis, lipid metabolism, and energy balance in the context of MetS.

Current evidence supports the involvement of major gut microbiota-associated mediators, including SCFAs, BAs, LPS, TMAO, and BCAAs, influencing host metabolic and inflammatory pathways. However, microbial taxonomic signatures remain highly heterogeneous across populations and metabolic phenotypes, reflecting the influence of dietary patterns, host physiology, disease stage, and methodological differences. Despite this variability, different microbial communities may converge on similar functional outputs through metabolic redundancy, suggesting that microbiota-associated mediators offer a functional perspective for understanding microbiota–host metabolic communication.

A deeper understanding of these microbiota–mediator–host signaling networks may facilitate the identification of potential intervention targets. Given the multifactorial nature of MetS, integrating microbiota-directed strategies with established lifestyle interventions, including dietary modification, caloric control, and increased physical activity, may provide new opportunities for developing more personalized adjunctive approaches for MetS management.

## Figures and Tables

**Table 1:** Microbial features associated with metabolic syndrome phenotypes.

Metabolic Phenotype	Reported Microbial Alterations	Associated Microbial Functions	Frequently Reported Taxa	Ref.
Obesity	Altered microbial diversity; Variable F/B ratio; Altered abundance of SCFA-producing bacteria.	Energy metabolism; SCFA and BA metabolism; Intestinal barrier regulation; Inflammatory signaling.	Enriched: *Dorea*, *Collinsella*, *Desulfovibrio*. Depleted: *Faecalibacterium*, *Roseburia*, *Akkermansia*, *Bifidobacterium*.	[28,31,32,33,34]
Insulin resistance	Altered microbial diversity; Reduced abundance of butyrate-producing bacteria.	SCFA metabolism; Incretin regulation; BCAA metabolism; Carbohydrate metabolism; Intestinal barrier integrity; Inflammatory signaling.	Enriched: *Collinsella*, *Desulfovibrio*. Depleted: *Oscillibacter*, *Ruminococcus*, *Faecalibacterium*.	[35,36,37,38,39]
Dyslipidemia	Variable F/B ratio; Altered BA-associated microbial composition.	BA metabolism; FXR/TGR5-associated metabolic signaling.	Enriched: *Agathobacter.* Depleted: *Bifidobacterium*, *Ruminococcaceae*.	[40,41,42]
Hypertension	Altered microbial diversity; Enterotype variation; Altered abundance of SCFA-producing bacteria.	TMAO-related metabolism; SCFA metabolism; Inflammatory signaling.	Enriched: *Prevotella.* Depleted: *Bifidobacterium*, *Roseburia*, *Faecalibacterium*, *Bifidobacterium*.	[43,44]

Abbreviations: F/B = Firmicutes/Bacteroidetes; SCFAs = Short-chain fatty acids; BAs = Bile acids; TMAO = Trimethylamine N-oxide; BCAAs = Branched-chain amino acids; FXR = Farnesoid X receptor; TGR5 = Takeda G protein-coupled receptor 5.

## Data Availability

No new data were created or analyzed in this study. Data sharing is not applicable to this article.

## Abbreviations

The following abbreviations are used in this manuscript:

MetS	Metabolic Syndrome
IR	Insulin resistance
SCFAs	Short-chain fatty acids
BAs	Bile acids
LPS	Lipopolysaccharide
TMAO	Trimethylamine N-oxide
BCAAs	Branched-chain amino acids
FFAR2/3	Free fatty acid receptor 2/3
NLRP3	NOD-like receptor family pyrin domain-containing protein 3
NF-κB	Nuclear factor κB
GLP-1	Glucagon-like peptide-1
FXR	Farnesoid X receptor
TGR5	Takeda G protein-coupled receptor 5
mTORC1	Mechanistic target of rapamycin complex 1
CD14	Cluster of differentiation 14
FMT	Fecal microbiota transplantation
LBPs	Live biotherapeutic products
